# Phosphine‐incorporated Metal‐Organic Framework for Palladium Catalyzed Heck Coupling Reaction

**DOI:** 10.1002/open.202300249

**Published:** 2024-04-09

**Authors:** Wenmiao Chen, Insha Shaikh, Fatma Ahmed, Sahar Karkoub, Mamoun AlRawashdeh, Hongcai Zhou, Sherzod Madrahimov

**Affiliations:** ^1^ Department of Science Texas A&M University at Qatar Education City, P.O. Box 23874 Doha Qatar; ^2^ Department of Chemical Engineering Texas A&M University at Qatar Education City, P.O. Box 23874 Doha Qatar; ^3^ Department of Chemistry Texas A&M University College Station Texas 77843–3255 United States

**Keywords:** metal−organic framework, mono(phosphine)-Pd complex, post-synthetic modification, Heck cross-coupling reaction

## Abstract

As an emerging material with the potential to combine the high efficiency of homogeneous catalysts and high stability and recyclability of heterogeneous catalysts, metal‐organic frameworks (MOFs) have been viewed as one of the candidates to produce catalysts of the next generation. Herein, we heterogenized the highly active mono(phosphine)‐Pd complex on surface of UiO‐66 MOF, as a catalyst for Suzuki and Heck cross coupling reactions. The successful immobilization of these Pd‐monophosphine complexes on MOF surface to form **UiO‐66‐PPh_2_–Pd** was characterized and confirmed via comprehensive set of analytical methods. **UiO‐66‐PPh_2_–Pd** showed high activity and selectivity for both Suzuki and Heck Cross Coupling Reactions. This strategy enabled facile access to mono(phosphine) complexes which are challenging to design and require multistep synthesis in homogeneous systems, paving the way for future MOF catalysts applications by similar systems.

## Graphical abstract

An effective heterogeneous catalyst was prepared through heterogenizing the mono(phosphine)‐Pd complex on surface of UiO‐66 (**UiO‐66‐PPh_2_–Pd**), for Suzuki and Heck cross coupling reactions. This catalyst combines the high reactivity of organometallic compounds and high recyclability of MOFs.

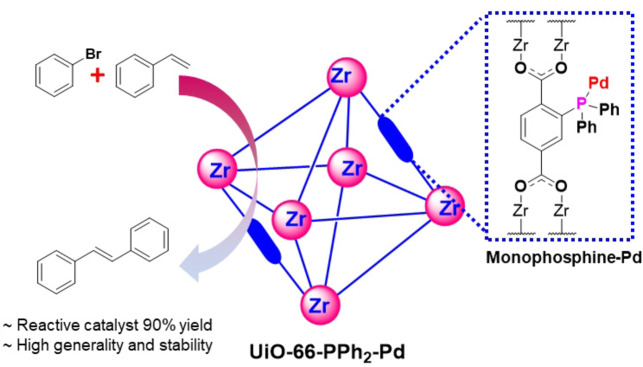

## Introduction

The rapid pace of industrialization over the last century, has caused numerous global energy and environmental concerns. This created a strong demand for technological innovations that involve search and discovery for versatile materials that meet both environmental and market needs in terms of sustainability and efficiency. The research in this area has led to development of materials that can serve as platforms for a myriad of applications.[^1^, ^2^, ^3^, ^4^, ^5^] Regarding catalytic systems, one method to address this problem is by immobilizing well‐known homogeneous catalysts on various supports. Homogeneous catalysts are the most active and selective systems for a majority of transformations due to their high activity and selectivity.[^6^, ^7^, ^8^, ^9^] The superior catalytic performance originates from the specially designed auxiliary ligands furnishing the metal center with the desired electronic and steric properties optimized for a specific reaction, which is hard to achieve in traditional solid supports like porous carbon and zeolite.[^10^, ^11^] For example, low coordinate complexes of metals including Pd are well known to be potent catalysts for C−C and C−N coupling reactions.[^12^, ^13^, ^14^] 12‐electron mono(phosphine) Pd complexes are especially reactive in these transformations.[^15^, ^16^, ^17^] However, the major challenge associated with homogeneous catalysts is their recyclability and relatively short lifetime under reaction conditions which hampers their use in industrial and medicinal applications.[^18^, ^19^, ^20^] Therefore, combining the strengths associated with homogeneous catalysts, such as high activity and selectivity with the recyclability and stability of heterogeneous catalysts can yield useful catalyst systems.

Metal‐Organic Frameworks (MOFs) are new class of materials, both functional and versatile, which received great popularity within academic and industrial research community due to its structural flexibility and practical properties.[^21^, ^22^, ^23^, ^24^] Through rational design o Because of these favorable properties, MOFs have been utilized in various green chemistry applications.[^25^, ^26^, ^27^, ^28^] MOF materials differ from inorganic materials in terms of their composition, morphology, and porosity that allow for rational immobilization of functional sites within their inorganic nodes (ions or clusters) and the organic linker.[^29^, ^30^] The MOF immobilized catalysts prepared this way combine the advantages of organometallic catalysts such as high activity and selectivity and heterogeneous catalysts such as stability and reusability.[^31^, ^32^, ^33^, ^34^, ^35^] Moreover, the flexible nature of MOFs endow them with the advantage to support multiple active species (single atom, nanoparticle) and achieve various functionalities.[^36^, ^37^, ^38^]

Numerous examples were reported using MOF‐based materials for Heck and Suzuki‐Miyaura cross coupling reactions, in which strategies like nanoparticle encapsulation, postsynthetic modulation (PSM) and reticular chemistry were applied.[^39^, ^40^, ^41^, ^42^, ^43^, ^44^, ^45^] Among them, construction of abovementioned underligated phosphine complex in MOFs is a promising solution harvesting the strength of well explored organometallic catalysts. Immobilization of the monodentate phosphine ligands on MOF materials had been utilized as an alternative method to access the low coordinate complexes and these catalytic materials have been applied for reactions such as hydrosilylation, hydrogenation and C−H borylation.[^33^, ^34^, ^35^]

Herein, we report the facile immobilization of a monodentate phosphine complex in MOF and subsequent application for Suzuki‐Miyaura and Heck cross coupling reactions.^[46]^ The MOF‐immobilized mono(phosphine) Pd catalyst was prepared from **UiO‐66** via ligand substitution followed by metalation. Compared with complicated pre‐synthesis or protection strategy in organometallic chemistry, our method through PSM is able to fabricate the mono‐(phosphine)–metal complexes directly on MOF with proper geometry.[^15^, ^33^, ^34^] This surface anchored monophosphine Pd(0) complex and its precursor were characterized via various structural analysis methods such as NMR, PXRD, XPS, SEM etc. The catalyst was utilized for Heck coupling reaction with activity and generality comparable to homogeneous complexes.[^47^, ^48^] The surface decorated sites avoid common deactivation paths like demetallation and dimerization. The synthesized **UiO66‐PPh_2_‐Pd** is reusable for at least four catalytic runs. It is also active for Suzuki couplings between aryl bromides and aryl boronic acids. This work highlights the MOF's strength for facile construction of surface organometallic catalysts for sustainable and recyclable organic synthesis.

## Results and Discussion

The Metal‐Organic Framework, **UiO‐66**, was synthesized according to previous reports under solvothermal conditions (S2.1, supporting information).[^32^, ^49^] According to this report, a mixture of zirconyl chloride octahydrate and organic linker, namely BDC (terephthalic acid) was heated at 90 °C for 18 hours with acetic acid as the modulating reagent in *N,N*‐dimethylformamide (DMF), yielding white powder. **UiO‐66** possesses a classical *pcu* topology where the 12‐connected Zr_6_ cluster is linked to 12 BDC molecules, forming a cubic open pocket. This high connectivity of Zr cluster endows the structure with ultrahigh stability yet relative low accessibility of the pore, in which the pore size is 6 Å.^[50]^ Thus, the decorated phosphine complexes are mainly on the surface of the MOF particles.

**Figure 1:**
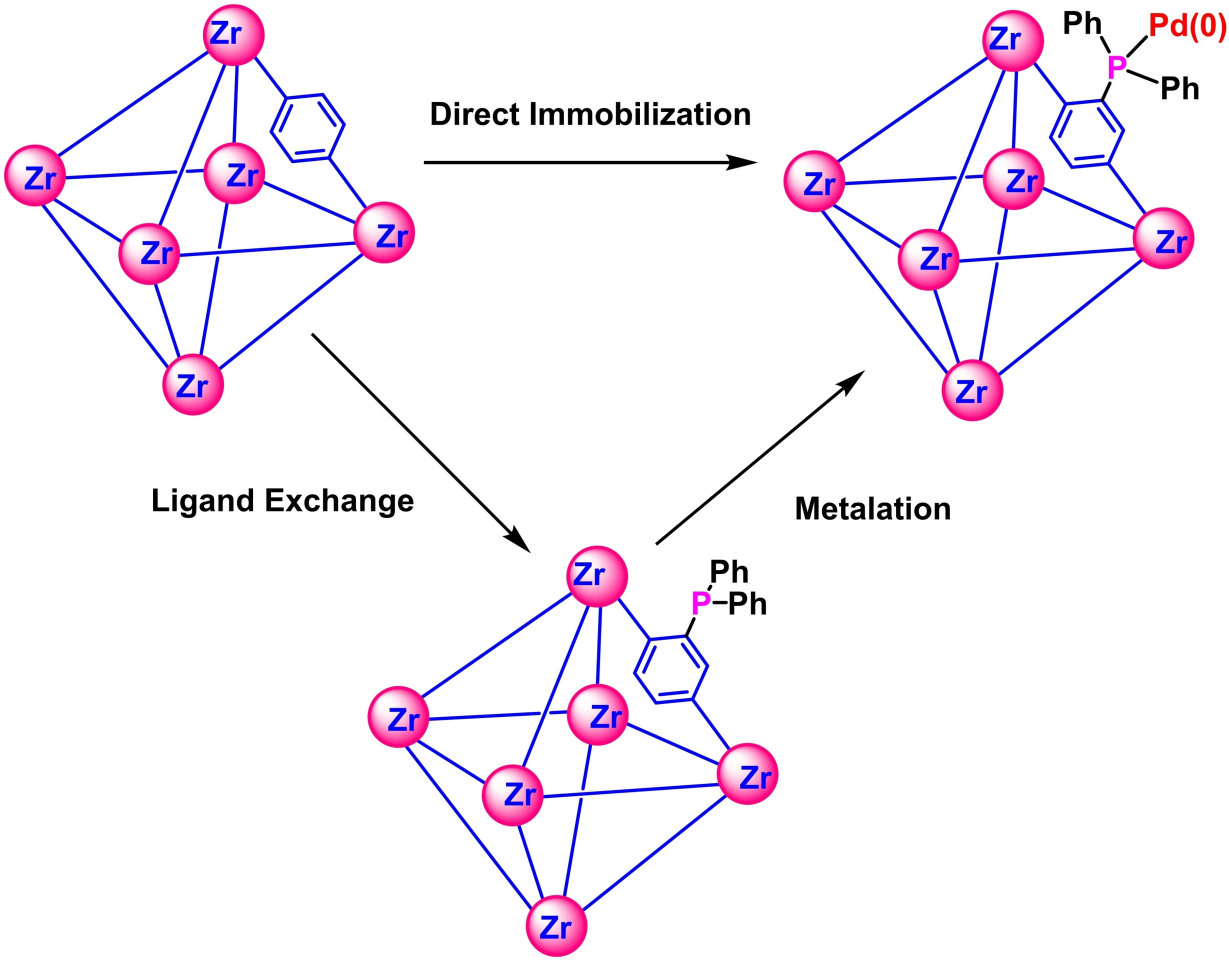
Schematic representation of the synthesis of **UiO66‐PPh_2_‐Pd** through post‐synthetic ligand exchange and metalation method.

**Figure 2:**
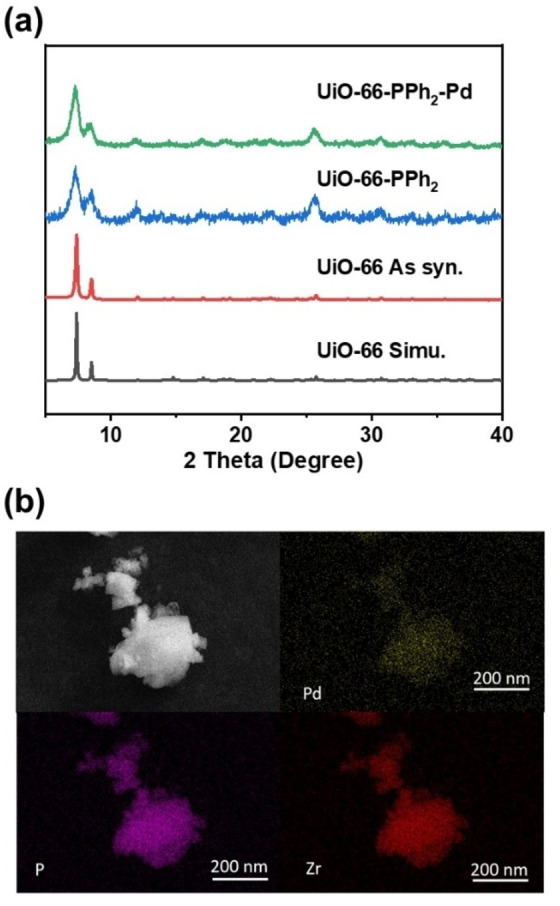
(a) PXRD pattern of **UiO‐66, UiO‐66‐PPh_2_** and **UiO66‐PPh_2_‐Pd** (b) EDS elemental mapping pictures of **UiO66‐PPh_2_‐Pd**.

The post‐synthesis linker exchange of the MOF followed by metalation affords the mono(phosphine)‐Pd complex (Figure 1). The ligand exchange was carried out by soaking the MOF samples in the DMF solution of monophosphine BDC‐PPh_2_ ligand as described in the SI, section S2.2. The successful exchange of ligand on the MOF surface was corroborated by a combination of solution and solid‐state nuclear magnetic resonance (NMR) methods. The MOF sample after ligand exchange was digested in concentrated D_2_SO_4_ for ^31^P NMR analyses (**Figure S1**, SI), which showed single phosphine peak at 34.3 ppm. This was ascribed to the phosphine oxide, which was unavoidable during digestion under the treatment of heat and acid.^[51]^ The partial oxidation is also observed in the commercial BDC‐PPh_2_ ligand (**Figure S2**, SI), which reduced the available coordination sites but caused no negative impact on the structure of framework. To confirm the nature of phosphine ligand grafted on MOF, solid‐state NMR (SS NMR) of ^31^P was conducted and showed a phosphine peak at −6 ppm, confirming the presence of immobilized phosphine ligands **(Figure S3a**, SI). Powder Xray diffraction (PXRD) of **UiO‐66‐PPh_2_** showed similar pattern to that of as synthesized **UiO‐66**, confirming the conservation of crystallinity during the ligand exchange (Figure 2a). The exchanged ratio was calculated based on the digested NMR with an external (phosphonic acid) standard in a capillary tube (Experiment details in S2.2, SI). According to the NMR, the calculated molecular weight is 1700 g/mol (calculated in one unit cell), corresponding to the formula Zr_6_O_4_(OH)_4_(BDC)_4.9_‐(BDC‐PPh_2_)_1.1_.

**UiO‐66‐PPh_2_** obtained via ligand exchange was metallated by soaking in solution of Pd(OAc)_2_ in methanol at room temperature (see SI, section S2.3). PXRD pattern of **UiO66‐PPh_2_‐Pd** (Figure 2a) shows the retention of the framework crystallinity after Pd immobilization. Scanning electron microscopy analysis and the energy‐dispersive spectroscopy (EDS) mapping of **UiO66‐PPh_2_‐Pd** after thorough wash (three times with acetone) showed no aggregation of Pd nanoparticles, revealing homogeneity with respect to the shape and size of the microcrystalline particles (Figure 2b). Moreover, no sign of aggregated Pd(0) nanoparticles is observed after comparing the XRD standard, confirming the atomic dispersion by monophosphine complexes (**Figure S4**, SI). Also, Pd valence was characterized by X‐ray photoelectron spectroscopy (XPS). The main peaks at 335 and 340.9 eV are assigned to the divalent Pd 3d_5/2_ and Pd 3d_3/2_ binding energy, showing that the Pd(O) is anchored on the phosphine ligands (**Figure S5a**, Supporting Information).^[47]^ XPS spectra of 2p orbital of P element show positive shift of 0.8 eV, indicating strong donating effect towards the Pd (**Figure S5b**). Similarly, the ^31^P‐SS NMR peak shifted to 60.7 ppm corresponding to the P−Pd(0) complex, with a small residual peak for the non‐coordinated phosphine oxide (**Figure S3b**, SI).^[52]^ Through inductively coupled plasma‐mass spectroscopy (ICP‐MS) analysis, the Pd/ Zr mole ratio of the digested **UiO66‐PPh_2_‐Pd** was at the range of 13 %, which afforded the molecular formula of Zr_6_O_4_(OH)_4_(BDC)_4.9_(BDC‐PPh_2_)_1.1_Pd_0.8_, showing that the Pd number is slight lower than phosphine ligands. As calculated molecular weight for the catalyst is about 1800 g/mol (calculated for one unit cell). These analytical results are consistent with immobilization of monodentate Pd phosphine on the MOF surface as single molecular species.

It is noteworthy that this post‐synthetic ligand exchange and metalation method enables access to the monophosphine complex on the MOF surface. In homogeneous phosphine ligand design, underligated or heteroleptic complexes are always achieved with the help of highly bulky substituents to stabilize the coordinatively unsaturated structure.^[53]^

After structural characterization, we investigated the catalytic activity of **UiO66‐PPh_2_‐Pd** towards the Suzuki−Miyaura and Heck cross coupling reactions. For the Suzuki coupling reaction of bromobenzene with phenylboronic acid, the reactions were catalyzed by 2 mg (0.5 mol % Pd) mass loading of **UiO66‐PPh_2_‐Pd** (Experimental details in S2.4, SI). Specifically, optimization of the catalytic activity with respect to solvent, temperature and choice of base was performed (see **Table S1**, SI). Optimal results were obtained in toluene at 100 °C, with 0.5 mol % Pd‐immobilized MOF catalyst loading and 1.5 *equiv*. of K_3_PO_4_ base. Using these conditions, the yield of the coupling product as observed by GC‐FID analysis is 93 %. (**Table S1**, entry 2, SI). This yield is comparable to those observed for homogeneous ligand‐Pd complexes (Pd with BDC‐PPh_2_), (93 % vs 90 % entries 2 and 7, **Table S1**, SI).

The prepared catalyst was also applied for Heck coupling reactions. Unlike Suzuki coupling reactions, this reaction was loaded in the glovebox with bromobenzene, styrene, base and catalyst. (**Table S2**, SI). As anticipated, **UiO66‐PPh_2_‐Pd** also showed high reactivity of 92 % yield under optimized condition, leading to a turnover frequency (TOF) of 15.3 (toluene as solvent and K_3_PO_4_ as base at 110 degree for 12 hours). Control experiments lacking metal, MOF or ligand were conducted (**Table S2**, entry 7, 8, 9), results of which were consistent with catalysis by Pd monophosphine complexes immobilized on the MOF surface. The heterogeneity of the catalyst was confirmed through the “hot filtration” test.^[45]^ In this test, it was observed that the reaction stopped at ~50 % conversion after removing the heterogeneous catalyst by centrifugation (**Figure S6**, SI). The catalyst exhibits good stability which can be recovered and reused 4 times with no loss of reactivity (**Figure S7**, SI). This gives out a turnover number (TON) of at least 61. PXRD and SEM before and after 4 runs show no sign of crystalline Pd(0), which is in line of EDS mapping, indicating no agglomeration or aggregation (**Figure S8** and **Figure S9**). It is in consistent with the TEM image, in which the catalyst shows no Pd nanoparticles after catalysis (**Figure S10**). Moreover, from the ICP measurement of the reaction solution as well as the used catalyst we found no evidence of Pd leaching and the Pd to Zr atomic ratio remained unchanged (13 %) compared with the original catalyst (**Table S3**, SI). Mechanistic estimation is made based on the Pd(0) to Pd(II) cycle, where a classic 4‐step reaction mechanism is drawn in **Figure S11**.^[19]^

The systemic study of the generality of **UiO66‐PPh_2_‐Pd** was conducted under optimized condition (Table 1). To our delight, **UiO66‐PPh_2_‐Pd** catalyzed the coupling reactions of styrene with a series of aryl bromide substrates with a range of electron withdrawing or electron donating substituents, all with excellent yields (Table 1). The yields are all excellent (around 90 %) regardless of the electronic effect of the substituents. For example, we observed similarly high isolated yields for aryl bromides with methyl (Table 1, b), methoxy (Table 1, c, f), and amine (Table 1, d) electron‐donating substituents, as we did with substituted aryl bromides with electron‐withdrawing groups such as aldehyde (Table 1, e), nitro (Table 1, g), and nitrile (Table 1, h). It is noteworthy to mention that the steric effect was not influential as it is normally the case for MOF immobilized catalysts as observed for the reaction of aryl bromide with a sterically bulky *meta*‐methoxy substitution provided 70 % yield (Table 1, f). The reduced yield observed for 1,4‐dibromobenzene (40 % for entry i) is ascribed to low catalyst loading for the disubstitution reaction. Furthermore, various substituted styrenes are tested. Both electron‐donating (Table 1, j, k) and electron‐withdrawing 4‐cyanostyrene (Table 1, l) exhibit high isolated yield, indicating the generality of the catalyst.

**Table 1:** Substrate scope of **UiO66‐PPh_2_‐Pd** Catalyst for Heck Coupling Reactions^a,b^.

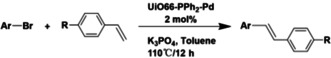			
entry	substrate	product	yield^b^
**a**	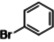	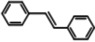	**92**
**b**	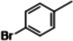	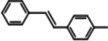	**90**
**c**	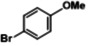	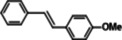	**90**
**d**	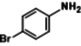	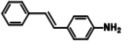	**91**
**e**	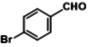	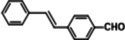	**93**
**f**	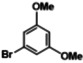	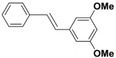	**70**
**g**	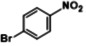	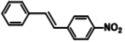	**89**
**h**	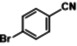	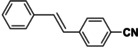	**91**
**i**	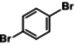	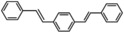	**40**
**j**	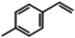	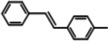	**92**
**k**	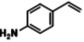	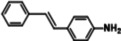	**90**
**l**	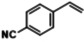	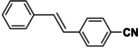	**88**

a. Reaction conditions: aryl bromide (0.2 mmol), styrene (0.3 mmol), toluene (2 mL), K_3_PO_4_ (0.3 mmol) and UiO66‐PPh_2_‐Pd (0.5 mol %). b. All yields are average of isolated yields over two runs. c. Entry i, 1,4‐dibromobenzene was reacted for 24 hours for full conversion.

## Conclusions

In summary, a novel method of surface immobilization of a catalytically active molecular complex on a MOF scaffold through post‐synthetic ligand exchange and metalation was developed. In contrast to homogeneous phosphine complexes, this surface decorated Pd complex is coordinatively unsaturated without the need for bulky ligands. This work demonstrates the utility of microporous MOFs which have high stability but limited application because of small pore size. With high reactivity for Suzuki coupling reaction (93 % yield), Heck coupling reaction (90 % average yield) and high stability of **UiO66** (90 % yield after 4 recycling), **UiO66‐PPh_2_‐Pd** should afford interesting opportunities for the design of potential highly active and stable MOF catalysts.

## Supporting Information

The Supporting Information is available free of charge on the ACS Publications website.

Detailed descriptions of synthetic procedures and other experiments as well as analytical data (PDF)

## Notes

The authors declare no competing financial interest.

## Conflict of interests

The authors declare no conflict of interest.

1

## Supporting information

As a service to our authors and readers, this journal provides supporting information supplied by the authors. Such materials are peer reviewed and may be re‐organized for online delivery, but are not copy‐edited or typeset. Technical support issues arising from supporting information (other than missing files) should be addressed to the authors.

Supporting Information

## Data Availability

The data that support the findings of this study are available in the supplementary material of this article.
