# Supplementary material for: Phosphine‐incorporated Metal‐Organic Framework for Palladium Catalyzed Heck Coupling Reaction
Source: ChemistryOpen. 2024 Apr 9;13(8):e202300249. doi: 10.1002/open.202300249 (PMC11319216; doi:10.1002/open.202300249)
Supplement: Supplementary file 1 — Supporting Information [file OPEN-13-e202300249-s001.pdf]

ChemistryOpen

Supporting Information

Phosphine-incorporated Metal-Organic Framework for Palladium Catalyzed Heck Coupling Reaction

Wenmiao Chen,* Insha Shaikh, Fatma Ahmed, Sahar Karkoub, Mamoun AlRawashdeh, Hongcai Zhou,* and Sherzod Madrahimov*

Supporting Information

Phosphine-incorporated Metal-Organic Framework for Palladium Catalyzed Heck Coupling Reaction

Wenmiao Chen[1,3]*, Insha Shaikh[2], Fatma Ahmed[2], Sahar Karkoub[2], Mamoun AlRawashdeh[2], Hongcai Zhou[3*], Sherzod Madrahimov[1]*

1 Department of Science, Texas A&M University at Qatar, Education City, P.O. Box 23874, Doha, Qatar

sherzod.madrahimov@qatar.tamu.edu

2 Department of Chemical Engineering, Texas A&M University at Qatar, Education City, P.O. Box 23874, Doha, Qatar

3 Department of Chemistry, Texas A&M University, College Station, Texas 77843-3255, United States

cwm-tamu@tamu.edu; zhou@chem.tamu.edu

S1. General Information.

All reactions involving air and moisture sensitive compounds were carried out in a glovebox under argon atmosphere. All reagents and solvents were commercially available and used as supplied without further purification, unless otherwise noted. All glassware was oven-dried before use.

PXRD was carried out with a Bruker D8-Focus Bragg–Brentano X-ray Powder Diffractometer equipped with a Cu sealed tube (λ=1.54178 Å) at 40 kV and 40 mA. SCXRD was measured on a Bruker Venture CMOS diffractometer equipped with a Cu-Kα sealed-tube X-ray source (λ=1.5406 Å).

ICP-MS data were collected with a Perkin Elmer NexION 300D ICP-MS.

TGA was conducted on a TGA-50 thermogravimetric analyser.

Field-emission SEM images were collected on the FEI Quanta 600 field-emission SEM at 20 KV. Source: Field emission gun assembly with Schottky emitter source. Beam Current: >100 nA.

The high resolution XPS measurements were performed with a Perkin Elmer PHI system. The sample was placed into the XPS chamber with a base pressure of <1.0 × 10−9 Torr. Al Kα (1486.6 eV) X-ray source at a chamber was used to excite photoelectrons. The spectra were recorded by using a 16-channel detector with a hemispherical analyzer.

^{1}H, ^{13}C and ^{31}P NMR spectra were recorded on a Bruker Avance 400 spectrometer and referenced to the residual solvent peak. ^{1}H NMR data are reported as follows: chemical shift (multiplicity (bs = broad singlet, s = singlet, d = doublet, t = triplet, q = quartet, p = pentet and m = multiplet), coupling constant and integration). ^{1}H, ^{13}C and ^{31}P NMR chemical shifts are reported in ppm downfield from tetramethylsilane (TMS, δ scale) using the residual solvent resonances as internal standards.

^{31}P NMR details:

To get quantitative ratio of compound with external standard from the peak ratio. ^{31}P NMR was measured at 400MHz, and 1024 scans, relaxation delay 4 s, an acquisition time of 0.498 sec.

S2. Experimental Section.

S2.1. Synthesis of UiO-66.

UiO-66 size of 14 nm was synthesized according to a previous procedure published by Morris et al.[1] Benzene-1,4-dicarboxylic acid (500mg, 2.4 mmol) was dissolved in 10 mL of *N,N* Dimethylformamide (DMF). In a separate vial, zirconyl chloride octahydrate (210 mg, 0.66 mmol) was dissolved in 30 mL of DMF. After sonication, the solutions were combined, and 3 mL acetic acid was added and further sonicated for 15 mins. The combined solution was heated in a temperature controlled oven at 90ºC for 18 hrs. Then the white jelly-like MOF nanoparticle was purified by centrifugation at 6000 rpm for 20 minutes followed by solvent exchange (3 x DMF and 3 x Acetone) over a 24 h period to afford white MOF powder. The nanoparticles were weighted and collected with the yield of 73% (calculated from $ZrCl_4$).

S2.2. Synthesis of UiO-66- PPh_2 through ligand exchange

The **UiO-66-PPh_2** ligand exchanged MOF was obtained using 250 mg of **UiO-66** and 100 mg of 2 -(diphenyl phosphino) terephthalic acid (BDC-PPh_2) in a vial. 25 mL of DMF was added to form a solution mixture and was sonicated for 1.5 hrs. A magnetic stirrer was put in the flask and the reaction was stirred overnight. The solution was then washed with DMF twice and DMF was decanted after centrifuging the MOF/DMF mixture at 6000 rpm for 15 mins. To remove the uncoordinated or monodentate ligands, a solution of 20 mL DMF and 2 mL HCl was added and placed in the oven for 6 hours. It was then centrifuged

at 6000 rpm for 15 mins and the supernatant was decanted, followed by three more procedures of washing, centrifuging and decanting the supernatant, once with DMF and two times with acetone. Finally, it was dried in the oven overnight. A total of 250 mg **UiO-66-PPh$_2$** was obtained, with the total ligand conversion 51% (calculated from BDC-PPh$_2$).

Details of digested NMR with ^{31}P external standard:
Metal-organic framework (MOF) materials were digested for ^{1}H NMR and ^{31}P NMR analysis by sonicating a small amount of MOF material (~3 mg) in conc. D_2SO_4 (5 drops) until all of the MOF disintegrated and a brown heterogeneous solution formed. Upon addition of $(CD_3)_2SO$ (0.5 mL) to this solution and further sonication, a homogeneous pale yellow solution forms, which was analyzed by ^{31}P NMR spectroscopy. The molecular weight of **UiO-66-PPh$_2$** based on immobilized ligands was calculated by the ratio of the phosphine peak coming from the MOF solution (chemical shift 34.38 ppm) against the ^{31}P peak of phosphonic acid D_2O solution (chemical shift 0 ppm) of known concentration (0.01 mol/L) added as an external standard in a capillary tube. We note that it is important to have a homogeneous solution of acid digested MOF of sufficient concentration for accurate integration of the signals. Yet, unfortunately through digestion the oxidation of phosphine ligands is unavoidable under the condition of heat and acid treatment. Thus, the phosphine peak shifts from -5.6 to 34 ppm.

S2.3. Synthesis of UiO-66-PPh$_2$-Pd through metalation
To a mixture of **UiO-66- PPh$_2$** (107 mg) and Palladium (II) chloride (15 mg), 10 ml of methanol was added in a 20 mL vial and mixed uniformly at room temperature overnight. The solution changed from white to brown. Furthermore, the solution was washed several times with methanol until the liquid on top became colorless. The final wash was done with acetone. Finally, the **UiO-66-PPh$_2$-Pd** catalyst obtained was collected and stored in a vial as dark-brown powder. The nanoparticles were collected with the Pd conversion of 70% (calculated from $PdCl_2$).

S2.4. Catalytic activity of UiO-66-PPh$_2$-Pd.
Heck reaction:
In a typical run of Heck Coupling reaction reactivity test, 0.2 mmol bromobenzene (21 µL), 0.3 mmol styrene (30µL) and 0.29 mmol base were combined in a vial containing 2 ml toluene under argon gas. 2 mg of the synthesized catalyst, **UiO-66-PPh$_2$-Pd** (0.0011 mmol, 0.5 % Pd), was then transferred to the solution. The vial was incubated at 110 °C for 12 hours, followed by centrifugation to separate the solid. The yield was calculated from NMR spectra in chloroform-d_3 obtained after removing the volatiles under vacuum and adding 0.2 mmol mesitylene (28 uL) as internal standard.
A substrate scope screening was performed using the optimized condition: Using the glove box under argon gas, 2 mL of toluene, 2 mg of **UiO-66-PPh$_2$-Pd (**0.0011 mmol, 0.5 % Pd), 63.7 mg (0.3 mmol) K_3PO_4 was mixed with 30 µL (0.3 mmol) of styrene and 0.2 mmol of different bromo-substrates. Due to the covering of NMR peaks for some substituents with mesitylene, all substrate screening yields were calculated by pure compounds after isolation using column chromatography.
Suzuki-Miyaura reaction:
In a typical run of Suzuki Coupling reaction reactivity test, 0.2 mmol bromobenzene (21 µL), 0.2 mmol phenylboronic acid and 0.4 mmol base were combined in a vial containing 2 ml toluene. 2 mg **UiO-66-PPh$_2$-Pd (**0.0011 mmol, 0.5 % Pd) was then transferred to the solution. The vial was incubated at 110 °C for 12 h, followed by centrifugation to separate the solid. The yield was calculated from NMR spectra in

chloroform-d_3 obtained after removing the volatiles under vacuum and adding 0.2 mmol mesitylene (28 uL) as internal standard.

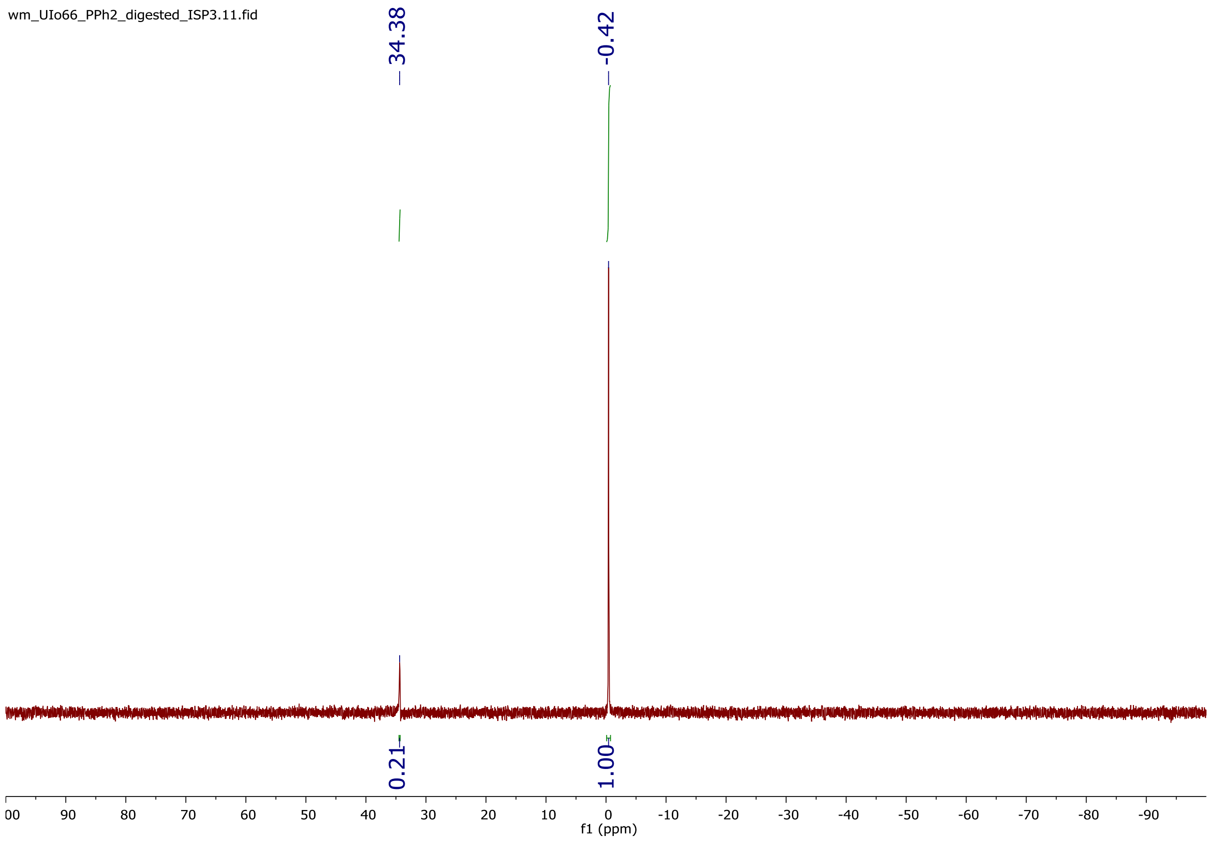

Figure S1. ^{31}P for the **UiO-66-PPh$_2$** digested in D_2SO_4/DMSO-*d6*. (Oxidized through hydrolysis) PPh2: shift 34.3ppm, integration 0.21, Formula: UiO-66-$(PPh_2)_{1.1}$

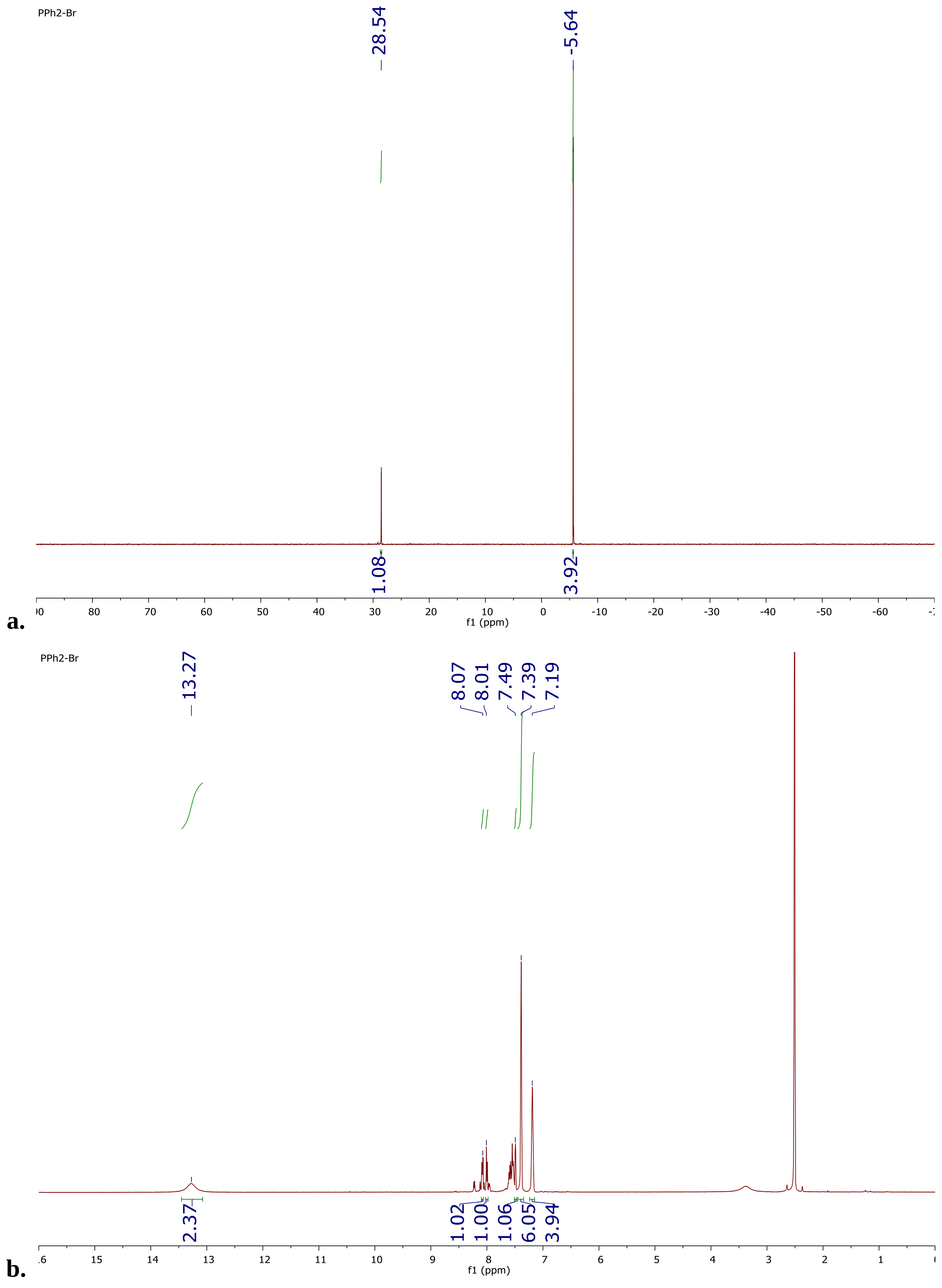

Figure S2. a. ^{31}P and b. ^{1}H NMR for the **BDC-PPh₂** ligand in DMSO-*d6* (partially oxidized).

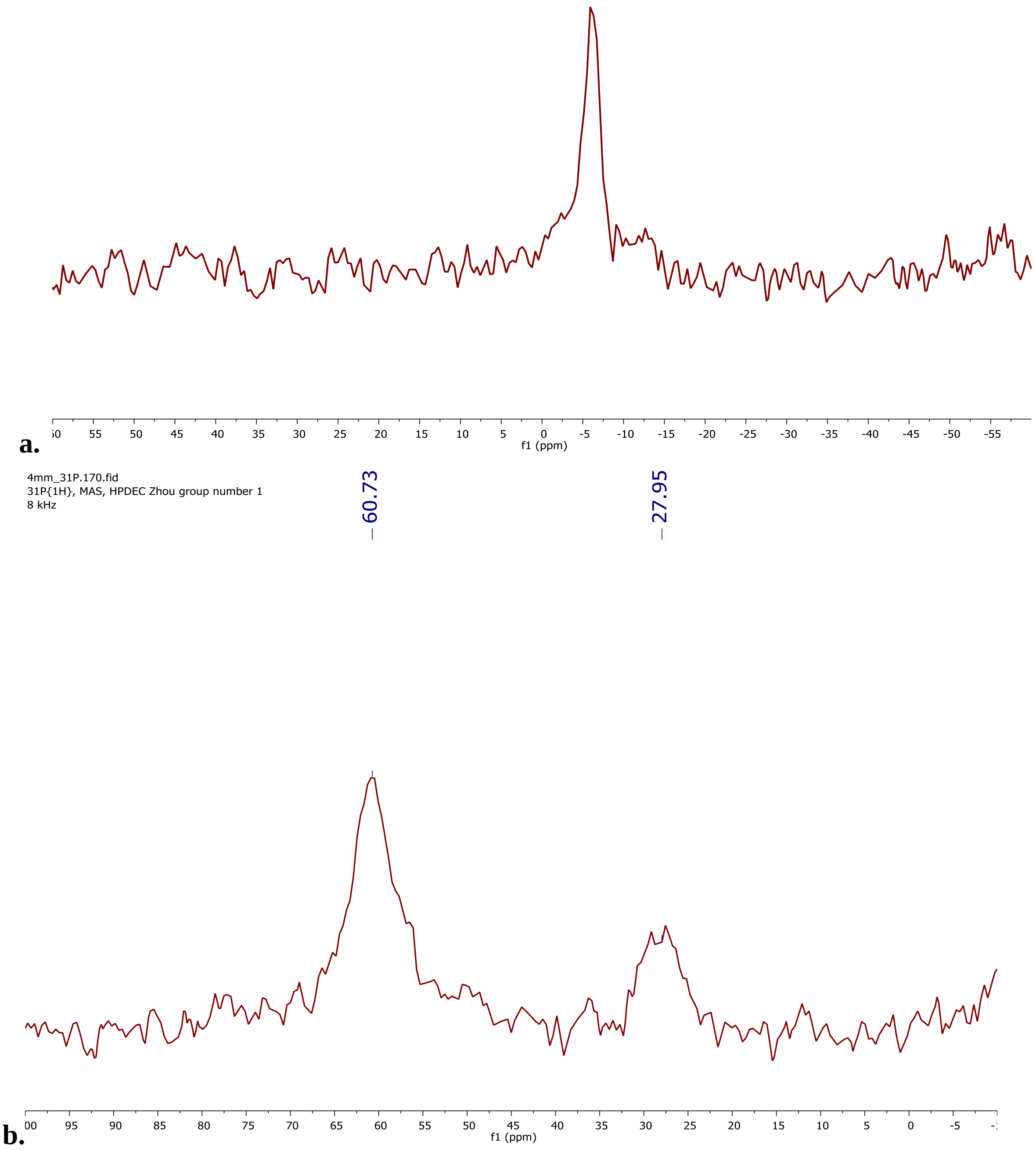

Figure S3. a. ^{31}P SSNMR for **UiO-66-PPh$_2$** and b. ^{31}P SSNMR for **UiO-66-PPh$_2$-Pd.**

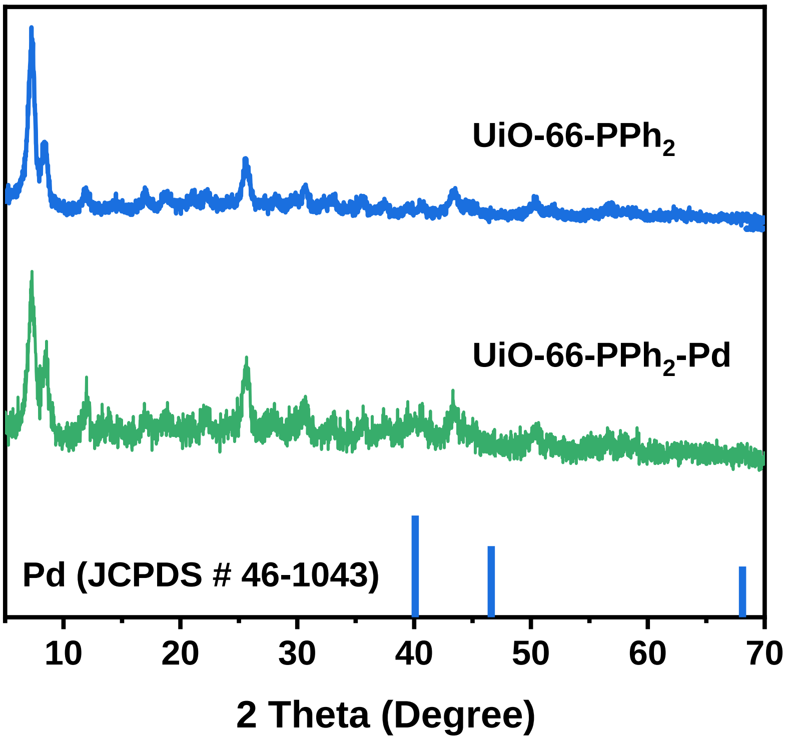

Figure S4 PXRD of **UiO-66-PPh$_2$, UiO-66-PPh$_2$-Pd** and face-centered cubic structure of Pd(0) (JCPDS no. 46-1043).

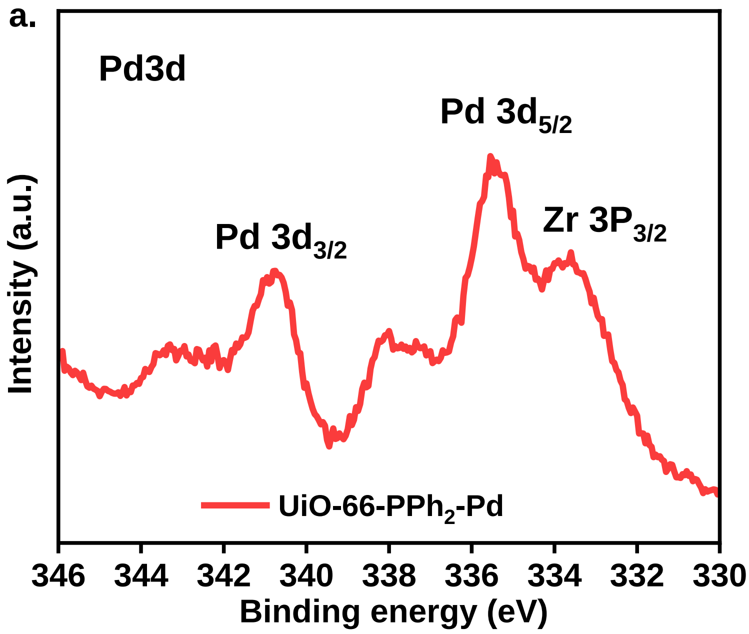

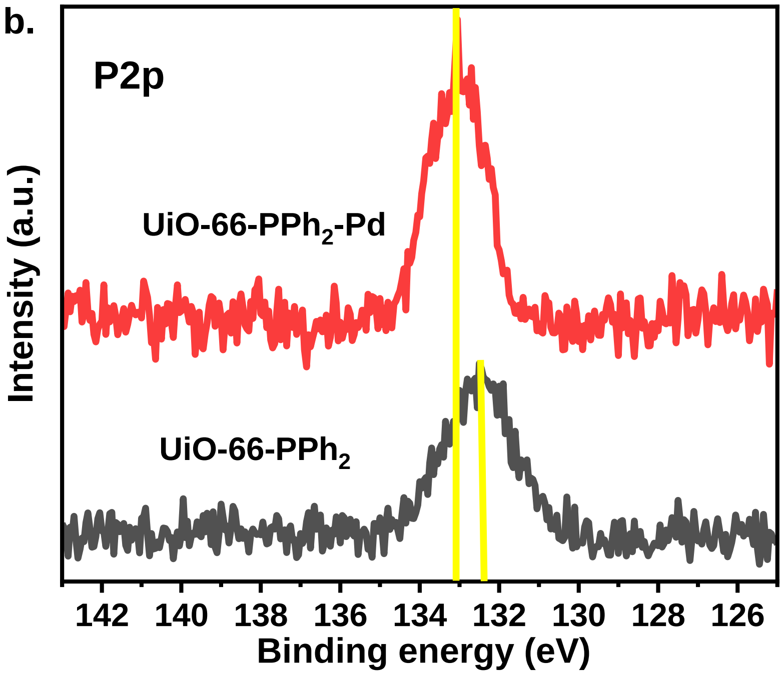

Figure S5. a. Pd3d and b. P2p spectrum of **UiO-66-PPh$_2$-Pd** from XPS analysis.

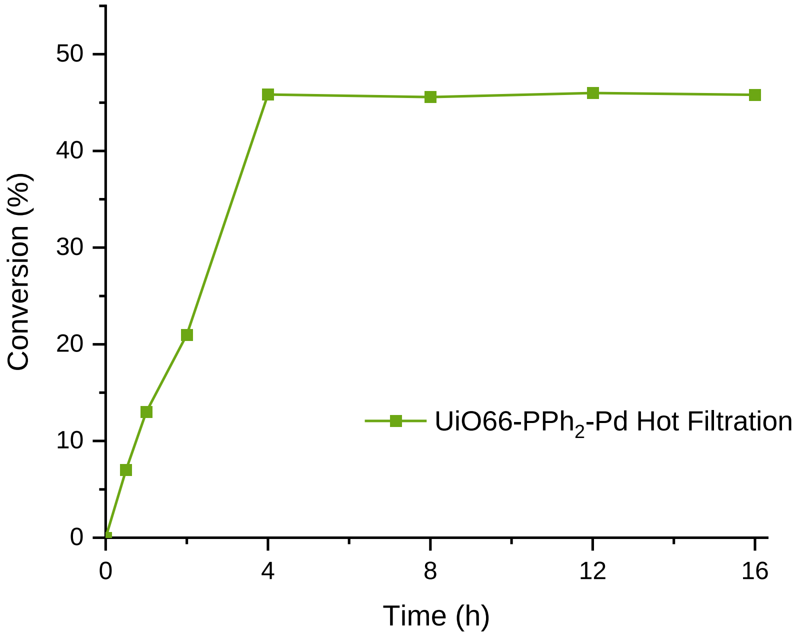

Figure S6. Hot filtration test of **UiO-66-PPh$_2$-Pd** for Heck cross-coupling reaction.

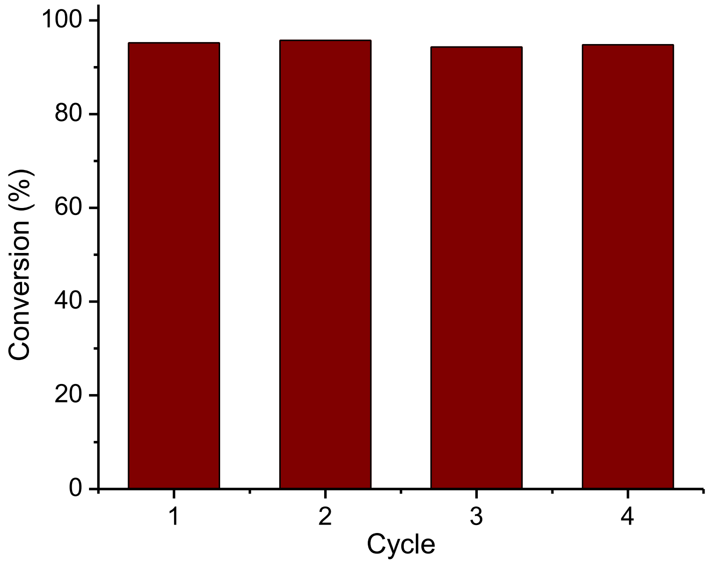

Figure S7. Recycling experiments of the Heck cross coupling reaction between bromobenzene and styrene conducted under optimized conditions.

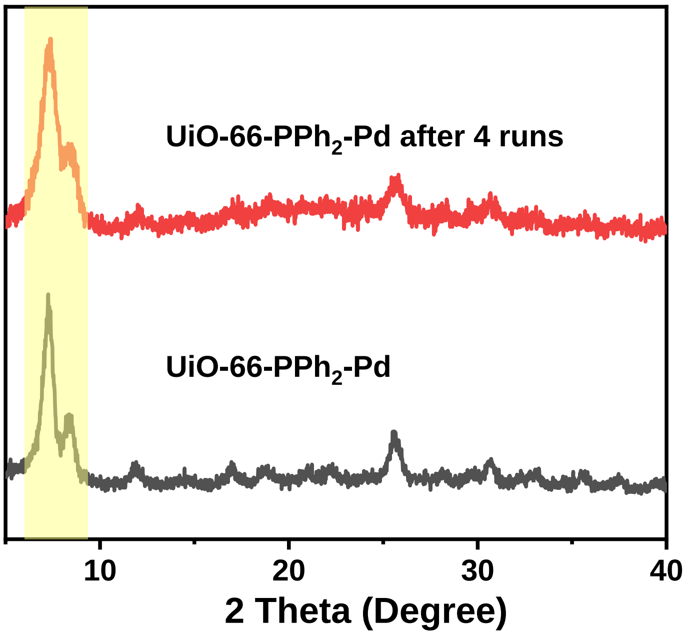

Figure S8. PXRD pattern of **UiO-66-PPh$_2$-Pd** and **UiO-66-PPh$_2$-Pd after 4 runs.**

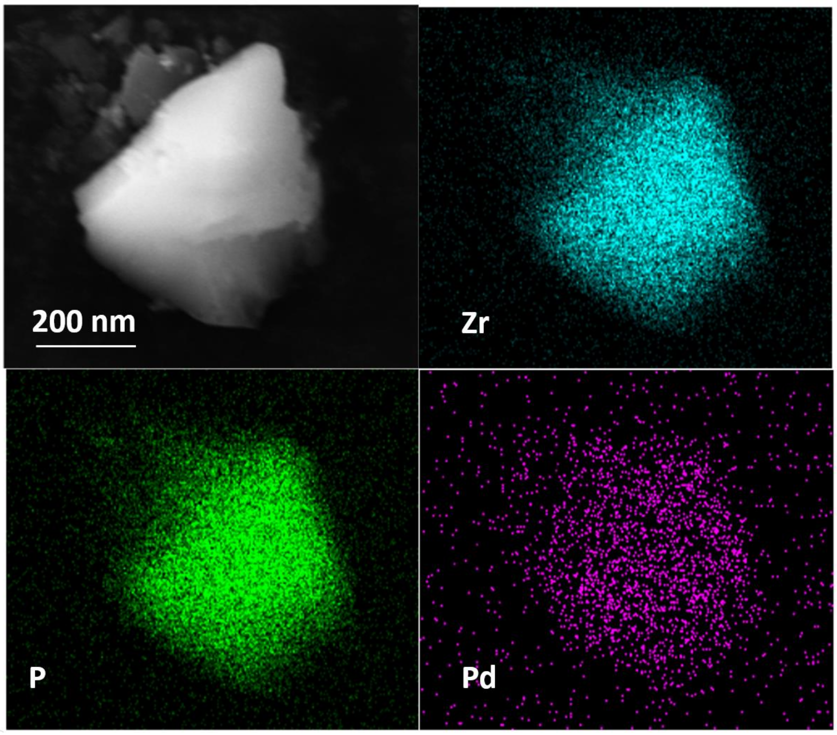

Figure S9. EDS elemental mapping pictures of **UiO66-PPh$_2$-Pd after 4 runs**.

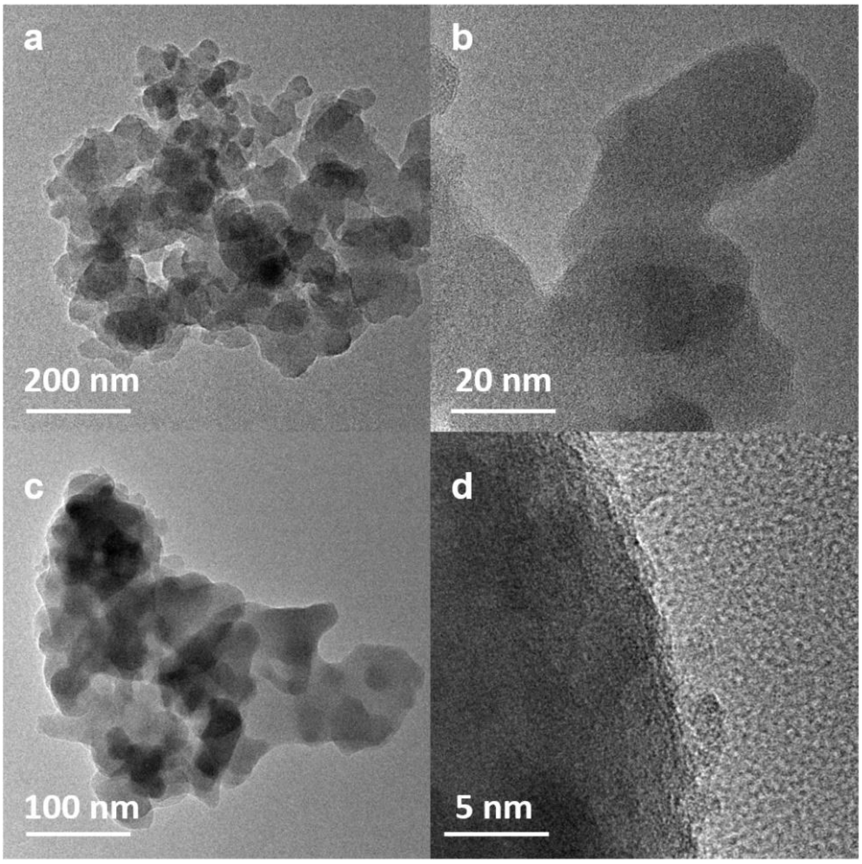

Figure S10. a, b TEM image of **UiO66-PPh$_2$-Pd** before catalysis and **a, b** TEM image of **UiO66-PPh$_2$-Pd** after 4 runs.

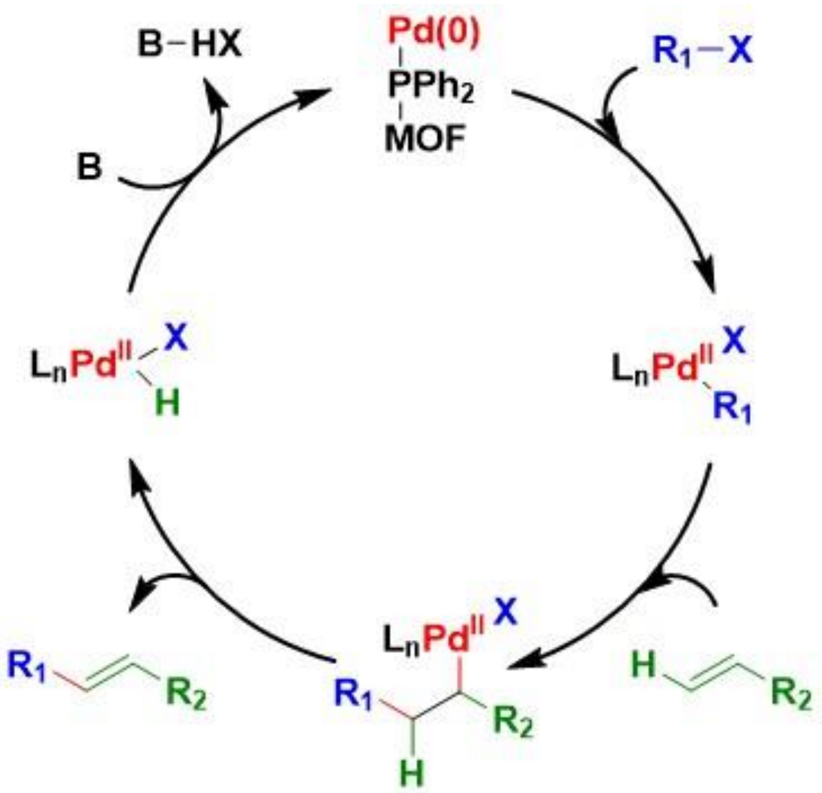

Figure S11. Proposed mechanism of Heck reaction catalyzed by **UiO66-PPh$_2$-Pd**. Catalytic species highlighted in red, aromatic halide in blue, aromatic alkene in green.

Table S1. Screening of **UiO-66-PPh$_2$-Pd** Catalyst for Suzuki-Miyaura Cross-Coupling Reactions[a]

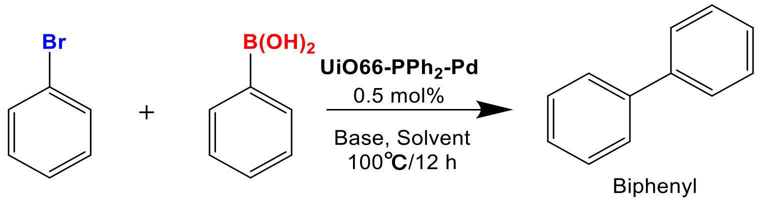

entry	cat.	solvent	base	yield[b]
1	UiO-66-PPh$_2$-Pd	toluene	K_2CO_3	80
2	UiO-66-PPh$_2$-Pd	toluene	K_3PO_4	93
3	UiO-66-PPh$_2$-Pd	DCM	K_2CO_3	N/A
4	UiO-66-PPh$_2$-Pd	MeCN	K_2CO_3	7
5	UiO-66Pd	DMF	K_2CO_3	16
6	UiO-66-PPh$_2$	toluene	K_2CO_3	N/A
7[c]	$PdCl_2$ + BDC-PPh$_2$	toluene	K_2CO_3	90

[a]Reaction conditions: bromobenzene (0.2 mmol), phenylboronic acid (0.2 mmol), solvent (2 mL), base (0.4 mmol), time (12 h), and **UiO-66-PPh$_2$-Pd** (0.5 mol %). [b]Yield was determined by GC-FID with mesitylene as internal standard. [c]Organic linker and metal salt was added instead.

Table S2. Screening of **UiO-66-PPh$_2$-Pd** Catalyst for Heck Cross-Coupling Reactions[a]

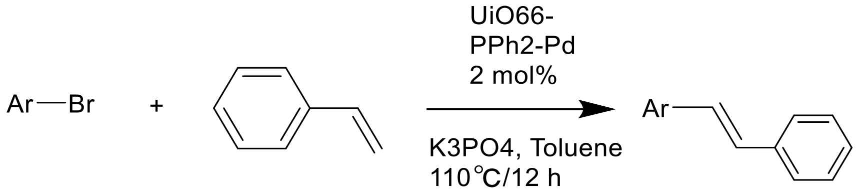

**entry**	**cate.**	**solvent**	**base**	**yield[b]**
**1**	UiO-66-PPh$_2$-Pd	toluene	K_3PO_4	92
**2**	UiO-66-PPh$_2$-Pd	toluene	K_2CO_3	75
**3**	UiO-66-PPh$_2$-Pd	toluene	NaOH	10
**4**	UiO-66-PPh$_2$-Pd	toluene	KOtBu	52
**5**	UiO-66-PPh$_2$-Pd	DCM	K_3PO_4	2
**6**	UiO-66-PPh$_2$-Pd	MeOH	K_3PO_4	12
**7**	UiO-66-PPh$_2$	toluene	K_3PO_4	N/A
**8[c]**	$PdCl_2$ + PPh$_2$ acid	toluene	K_3PO_4	42
**9[d]**	UiO-66 + $PdCl_2$	toluene	K_3PO_4	N/A
**10[e]**	UiO-66-PPh$_2$-Pd	toluene	K_3PO_4	90
**11**	UiO-66-PPh$_2$-Pd	toluene	K_3PO_4	90

[a]Reaction conditions: bromobenzene (0.2 mmol), styrene (0.3 mmol), solvent (2 mL), base (0.4 mmol), time (12 h), and UiO-66-PPh$_2$-Pd(2 mg, 0.5 mol %), the reaction was loaded in the glovebox. [b]Yield was calculated from isolated products over 2 parallel experiments. [c]Organic linker and metal salt was added instead. [d]Catalyst was prepared with $PdCl_2$ presursor. [e]Catalyst was recycled through centrifugation and reused for the second and third time.

Table S3. Pd to Zr ratio tested by the ICP-MS.

**Sample Name**	**Pd Ratio**	**Zr Ratio**
**UiO66-PPh$_2$-Pd**	**0.13**	**1**
**UiO66-PPh$_2$-Pd 4 runs**	**0.14**	**1**
**Reaction solution [a]**	**Not found**	**Not found**

a: Reaction solution after 4 runs was diluted and tested by ICP-MS, where no desolved Pd or Zr was found.

S3. Product Characterization.

Products Characterization for **UiO-66-PPh$_2$-Pd** Catalyzed Suzuki-Miyaura Cross-Coupling[2]

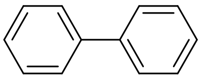

Biphenyl: White Solid. Column chromatography using Hexanes. ^{1}H NMR (400 MHz, $CDCl_3$), δ (ppm):

7.64 (d, 4H, H_{ar}), 7.47 (t, 4H, H_{ar}), 7.38 (t, 2H, H_{ar}). ^{13}C NMR (400 MHz, CDCl3), δ (ppm): 141.3 (s, C, C_{ar}), 128.8 (s, CH, C_{ar}), 127.3 (s, CH, C_{ar}), 127.2 (s, CH, C_{ar}).

Products Characterization for **UiO-66-PPh$_2$-Pd** Catalyzed Heck Cross-Coupling[3-4]

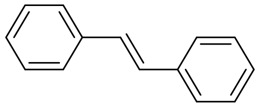

(E)-Stilbene (3a): White solid. Column chromatography using Hexanes. ^1H NMR (400 MHz, DMSO-D_6), δ: 7.60 (d, 4H, H_{ar}), 7.43 (t, 4H, H_{ar}), 7.32 (t, 2H), 7.19 (s, 2H, CH)

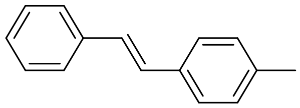

4-Methylstilbene: White solid. Column chromatography using Hexanes. ^1H NMR (400 MHz, DMSO-D_6) δ: 7.55 (d, 2H, H_{ar}), 7.46 (d, 2H, H_{ar}), 7.39 (d, 2H, H_{ar}), 7.33-7.28(m, 1H, H_{ar}), 7.21 (d, 2H, H_{ar}), 7.14 (d, 1H, CH), 7.09 (d, 1H, CH), 2.40 (s, 3H, CH3)

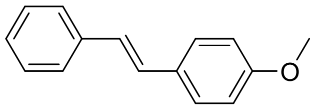

4-Methoxystilbene: White solid. Column chromatography using Hexanes. ^1H NMR (400 MHz, DMSO-D_6) δ: 7.36 (d, 2H, H_{ar}), 7.33 (d, 2H, H_{ar}), 7.26-7.22 (m, 2H, H_{ar}), 7.13-7.09 (m, 1H, H_{ar}), 6.94 (d, 1H, CH), 6.84 (d, 1H, CH), 6.76 (d, 2H, H_{ar}), 3.71 (s, 3H, OCH3)

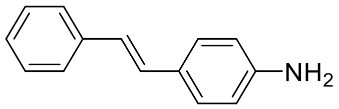

4-Aminostilbene: White solid. Column chromatography using Hexanes / AcOEt 1:1. ^1H NMR (400 MHz, DMSO-D_6) δ: 7.50 (d, 2H, H_{ar}), 7.32(d, 2H, H_{ar}), 7.14-7.21 (m, 2H, H_{ar}), 7.13-7.09 (m, 1H, H_{ar}), 6.92 (d, 1H, CH), 6.86 (d, 1H, CH), 6.57 (d, 2H, H_{ar}), 5.30 (s, 2H, NH2)

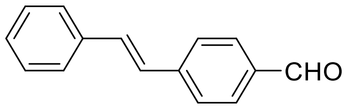

4-Formylstilbene: White solid. Column chromatography using Hexanes / AcOEt 2:1. ^1H NMR (400 MHz, DMSO-D_6) δ: 9.99 (s, H, CHO) 7.95 (d, 2H, H_{ar}), 7.90(d, 2H, H_{ar}), 7.83 (d, 2H, H_{ar}), 7.67 (d, 2H, H_{ar}), 7.51 (d, 1H, CH), 7.46 (d, 1H, CH), 7.34 (m, 1H, H_{ar}).

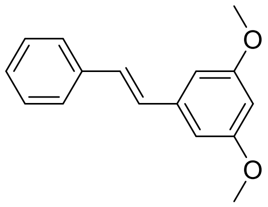

3,5-dimethoxystilbene: Colorless solid. Column chromatography using Hexanes / Dichloromethane 1:1 ^1H NMR (400 MHz, $CDCl_3$) δ (ppm) 7.71 (d, 2H, H_{ar}), 7.47 (t, 2H, H_{ar}), 7.37 (m, 1H, H_{ar}), 6.91 (d, 1H, CH), 6.93 (d, 1H, CH), 6.83 (d, 2H, H_{ar}), 6.51 (s, 1H, H_{ar}), 3.76 (s, 6H, CH_3).

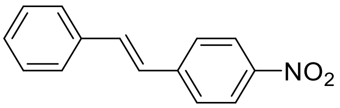

4-Nitrostilbene: Yellow solid. Column chromatography using Dichloromethane ^{1}H NMR (250 MHz, DMSO-D_6): δ=8.40 (d, J=9.3 Hz, 2H), 7.80 (d, J=9.3 Hz, 2H), 7.71 (d, J=7.3 Hz, 2H), 7.45–7.55 (m, 3H), 7.37 (d, J=16 Hz, 1H), 7.23 (d, J=16.5 Hz, 1H) ppm.

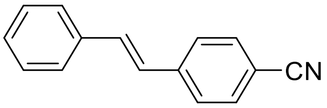

4-Cyanostilbene: Pale yellow solid. Column chromatography using Hexanes. ^{1}H NMR (400 MHz, DMSO-D_6): δ=7.45–7.60 (m, 6H), 7.49 (t, J=7.5 Hz, 2H), 7.40 (t, J=7.45 Hz, 1H), 7.20 (d, J=16.5 Hz, 1H), 7.02 (d, J=16.5 Hz, 1H) ppm.

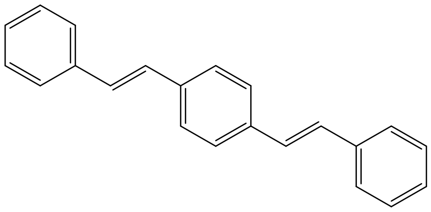

(Z,Z)-p-Styrylstilbene: : Colorless solid. Column chromatography using Hexanes. ^{1}H NMR (400 MHz, DMSO-D_6): δ: 8.71 (s, 4H, H_{ar}) 8.39 (d, 4H, H_{ar}), 8.15 (d, 4H, H_{ar}), 7.70 (t, 4H, H_{ar}), 7.59 (t, 2H, H_{ar}).

Reference:

(1) Morris, W.; Briley, W. E.; Auyeung, E.; Cabezas, M. D.; Mirkin, C. A. Nucleic Acid–Metal Organic Framework (MOF) Nanoparticle Conjugates. *J. Am. Chem. Soc.* **2014,** *136*, 7261-7264.

(2) Chen, W.; Cai, P.; Elumalai, P.; Zhang, P.; Feng, L.; Al-Rawashdeh, M. m.; Madrahimov, S. T.; Zhou, H.-C. Site-Isolated Azobenzene-Containing Metal–Organic Framework for Cyclopalladated Catalyzed Suzuki-Miyuara Coupling in Flow. *ACS Appl. Mater. Interfaces* **2021,** *13*, 51849-51854.

(3) Shang, N.; Gao, S.; Zhou, X.; Feng, C.; Wang, Z.; Wang, C. Palladium nanoparticles encapsulated inside the pores of a metal–organic framework as a highly active catalyst for carbon–carbon cross-coupling. *RSC Adv.* **2014,** *4*, 54487-54493.

(4) Iranpoor, N.; Firouzabadi, H.; Tarassoli, A.; Fereidoonnezhad, M. 1,3,2,4-Diazadiphosphetidines as new P–N ligands for palladium-catalyzed Heck reaction in water. *Tetrahedron* **2010,** *66*, 2415-2421.
